# 肥大细胞白血病合并嗜酸性粒细胞增多1例报告并文献复习

**DOI:** 10.3760/cma.j.issn.0253-2727.2022.05.012

**Published:** 2022-05

**Authors:** 玲洁 孙, 俊霞 黄, 茜茜 王, 方 侯, 宏 许, 洪国 赵, 伟 王, 海荣 费, 雪 史

**Affiliations:** 青岛大学附属医院血液科，青岛 266003 Affiliated Hospital of Qingdao University, Qingdao 266003, China

肥大细胞白血病（mast cell leukemia，MCL）是系统性肥大细胞增生症（systemic mastocytosis，SM）中罕见的一种类型。2016年的WHO造血与淋巴组织肿瘤分类将SM分为五种亚型[1]：惰性SM（indolent SM，ISM）、冒烟型SM（smoldering SM，SSM）、SM伴血液系统肿瘤（SM with associated hematologic neoplasm，SM-AHN）、侵袭性SM（aggressive SM，ASM）和MCL。MCL病情进展快，诊断困难，而合并嗜酸性粒细胞增多又增加了MCL诊断和治疗的难度。现报告我院新近诊断的一例MCL合并嗜酸性粒细胞增多患者并对相关文献进行复习，以提高对此罕见疾病诊疗的认识。

## 病例资料

患者，男，28岁，因“反复发热2个月余”于2020年11月30日入院。患者2个月余前进食辛辣食物后出现发热，伴腹痛、腹泻、骨痛，体温最高38 °C，伴面颈部及前胸皮肤潮红、球结膜充血、盗汗、乏力，于外院查血常规：WBC 13.3×10^9^/L，HGB 136 g/L，PLT 117×10^9^/L，嗜酸性粒细胞绝对计数（EOS）6.98×10^9^/L。胸腹部CT示脾大。骨髓象：分类不明细胞占8.5％，嗜酸性粒细胞多见，占2.98％。骨髓免疫分型：髓系原始细胞占1.92％，嗜酸性粒细胞比例明显增高。骨髓病理：骨髓增生大致正常，嗜酸性粒细胞增多。染色体核型正常。PDGFRA、PDGFRB、FGFR、IGH、IGK、TCRγ均未见异常，白血病融合基因筛查无异常。PET/CT检查：脾大，代谢均匀略增高，SUVmax约2.5，中轴骨及双侧肱骨、股骨近端弥漫性轻度代谢增高，SUVmax约3.5。院外诊断为嗜酸性粒细胞增多症，应用泼尼松15 mg每日2次，5 d后症状缓解，停药后患者症状出现反复，再次应用泼尼松15 mg每日3次，效果差，遂就诊我院。

入院查体：面部潮红，脾大，甲乙线10 cm，甲丙线12 cm，丁戊线-1 cm，质韧，无触痛。血常规：WBC 41.84×10^9^/L，HGB 112 g/L，PLT 40×10^9^/L，EOS 19.64×10^9^/L。可溶性CD25 30 306.78 pg/ml，血清类胰蛋白酶193.7 ng/ml，IL-5 76.98 pg/ml，IL-6 51.07 pg/ml。ENA酶谱、ANA、EBV-DNA、CMV-DNA均无异常。消化系统B超示：肝脾大，脾厚径8.1 cm，肋缘下10.6 cm，腹水。骨髓象：有核细胞增生明显活跃，分类不明细胞占28％，嗜酸性粒细胞多见，占35％（图1）。外周血涂片：分类不明细胞占14％。骨髓免疫分型：异常髓系细胞占有核细胞32.61％，该群细胞强表达CD117和CD33，表达CD13、CD123、CD22、CD4、CD9、CD25、CD64，弱表达CD2，考虑为肥大细胞增生症。嗜酸性粒细胞比例明显增高，占有核细胞的39.94％。骨髓病理：嗜酸性粒细胞比例明显增高，骨小梁间另见部分小圆细胞，核淡染，胞质弱嗜酸性，免疫组化示CD117（+）、MPO（−）、CD68（−）、CD34（−）、CD2弱（+）、CD15（−）、CD38（−）、Lysozyme（−），考虑为增生的肥大细胞（细胞数量大于15个）。诊断为MCL，给予氯雷他定10 mg/d、地塞米松10 mg/d改善组胺类物质相关症状和降低嗜酸性粒细胞。于2020年12月7日给予酪氨酸激酶抑制剂（TKI）+CLA方案诱导化疗（伊马替尼400 mg/d第1～12天，克拉屈滨10 mg/d第2～6天，阿糖胞苷100 mg/d第1～7天）。12月14日基因检测结果示KIT D816H突变阳性。考虑伊马替尼对KIT D816H突变无效，嘱患者家属联系购买米哚妥林。治疗期间患者出现感染、心功能不全、肺水肿、多浆膜腔积液，给予抗感染、利尿等对症支持治疗后好转。查体脾脏逐渐缩小至肋缘下未及，IL-5和IL-6逐渐降至正常。12月31日复查骨髓：分类不明细胞占2％，完全缓解（CR）。流式细胞术微小残留病（MRD）示：未见CD25^+^CD2^+^异常表型肥大细胞。未检测到KIT D816H突变。2021年1月1日开始应用米哚妥林50 mg每日2次。1月8日复查B超示：脾厚径6.5 cm，肋缘下未见。同日行CLA方案巩固化疗（克拉屈滨10 mg/d第1～5天，阿糖胞苷100 mg/d第1～7天）。1月27日复查骨髓象：原始细胞占1.5％，流式细胞术MRD：肥大细胞占有核细胞的0.45％，表型未见异常，WT1定量0.37％，未检测到KIT D816H突变。3月11日行父供子单倍体造血干细胞移植。+11 d粒系植活，+14 d复查骨髓形态仍CR，流式细胞术MRD阴性，WT1为0.06％。随访至2021年10月5日，疾病仍处于CR状态，流式细胞术MRD及基因检测均阴性。

**图1:**
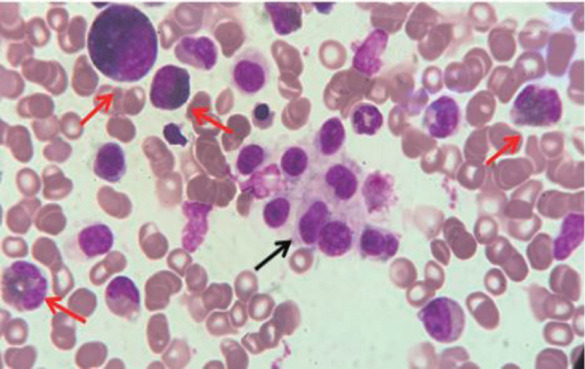
患者显微镜下骨髓细胞形态（×1000） 骨髓中可见一群分类不明细胞，该细胞胞体中等大小，核染色质较细致，核型不规则，胞质淡蓝色，部分可见颗粒，部分胞质边缘不整齐，呈云雾状（黑色箭头所指），嗜酸性粒细胞比例增高（红色箭头所指）

## 讨论及文献复习

MCL是SM的一种罕见类型，占所有SM不足1％，可以原发起病，也可以由其他SM亚型转化而来。2016年WHO关于SM的诊断标准包括主要标准：骨髓和（或）其他皮肤外器官可见多灶性、致密性肥大细胞浸润（聚集的肥大细胞数≥15个），和4项次要标准：①骨髓或皮肤外器官切片可见>25％的梭形或不典型形态肥大细胞浸润，或骨髓涂片中幼稚或不典型肥大细胞>25％；②骨髓、外周血或其他皮肤外器官中检测到KIT基因816密码子点突变；③骨髓、外周血或其他皮肤外器官中肥大细胞表达CD2和（或）CD25，同时也表达正常肥大细胞表面标志；④血清胰蛋白酶>20 ng/ml（有克隆性髓系肿瘤时该指标无效）。主要标准+1项次要标准，或满足3项次要标准，即可诊断SM[2]。各型诊断流程参见文献[2]。MCL临床表现差异性大，主要为与肥大细胞活化有关的症状（如面色潮红、发热、胸闷、全身乏力、腹痛腹泻、心动过速等）和器官受累的表现（如血细胞减少、胃肠道浸润、骨折、肝脾、淋巴结肿大等）[3]–[4]。诊断MCL除了满足SM的诊断标准外，还需满足：①骨髓活检示不典型的、幼稚的肥大细胞弥漫性浸润，常为间质性浸润；②骨髓穿刺涂片中肥大细胞≥20％；③外周血肥大细胞占白细胞总数的≥10％。若外周血肥大细胞比例<10％，则诊断为非白血病性MCL变异型[2]。本例患者有肥大细胞活化和浸润器官相关症状（面色潮红、发热、乏力、腹痛腹泻、脾大等），骨髓活检可见肥大细胞浸润（聚集的肥大细胞数≥15个），骨髓涂片肥大细胞大于25％，表达CD2、CD25和CD117，基因检测检出KIT 816密码子点突变，血清胰蛋白酶基础水平>20 ng/ml，外周血肥大细胞>10％。符合SM 1个主要及4个次要标准，同时也满足MCL的补充诊断，所以该患者MCL诊断明确。

MCL也是起源于干细胞的恶性克隆性疾病，其白血病性肥大细胞祖细胞存在于CD34^+^/CD38^−^细胞群中，同时表达CD33及CD133，将此类细胞移植入免疫缺陷小鼠中，可以诱导MCL发生[5]。MCL中肥大细胞免疫学检查表达CD9、CD25、CD133、CD117（KIT）以及CD2[6]–[7]，约三分之一的患者CD2/CD25双阴性[3]。目前报道的多数MCL患者染色体核型正常，但复杂核型、5q−、12p−、t（10；16）（q22；q13q22）以及t（8；21）（q22；q22）也可出现[3]–[4]。KIT D816位点的总体突变率在MCL患者中约为90％[3],[8]–[9]，大部分为KIT D816V位点突变，30％～50％为D816H、D816F、D816Y等其他位点突变。除此之外，MCL还可存在其他基因突变，如SRSF2、TEST2、TSXL1、N/KRAS、CBL、IDH1/2、ASXL1、RUNX1等，其中SRSF2/ASXL1/RUNX1（S/A/R）阳性提示预后不良[4]。

该患者以发热、腹痛、皮肤潮红起病，合并嗜酸性粒细胞增多。有文献报道高达28％的SM患者合并外周血嗜酸性粒细胞增多（>0.65×10^9^/L），晚期SM（AdvSM）中合并嗜酸性粒细胞增多的比例更高[10]，AdvSM包括SM-AHN、ASM和MCL。分析其机制，可能是因为嗜酸性粒细胞及肥大细胞均由CD34^+^祖细胞分化而来，在宿主免疫反应和维持机体正常稳态中发挥重要作用，二者可通过分泌可溶性介质或膜接触机制来相互影响。如肥大细胞产生的IL-3、IL-5能促进嗜酸性粒细胞的增殖和存活，其分泌的糜蛋白酶能够抑制嗜酸性粒细胞凋亡并诱导嗜酸性粒细胞释放IL-6。而IL-6、IL-3、IL-5这些细胞因子在干细胞因子的协同作用下，又能够促进肥大细胞的增殖和活化[11]。本例患者治疗前IL-5和IL-6 即明显升高，随着疾病的缓解，细胞因子逐渐降至正常。SM的嗜酸性粒细胞增多通常不是病理性的，患者的临床表现和靶器官损害往往是由增生的肥大细胞引起，而不是由克隆性嗜酸性粒细胞增多所致，因此通常不需要针对嗜酸性粒细胞特殊治疗，积极治疗SM原发病即可[12]。

MCL目前尚无统一的治疗标准。其治疗原则主要包括四大方面：①针对肥大细胞介质释放的治疗，如抗组胺药物的应用。此外，大剂量糖皮质激素最初被用于减轻改善肥大细胞活化症状，减轻腹水、疼痛、心力衰竭表现以及血细胞减少，但单用糖皮质激素效果不理想[13]。②减灭肥大细胞的治疗，如羟基脲、α干扰素、克拉屈滨等[14]。但α干扰素对AdvSM疗效差，不良反应多，无论是否联合糖皮质激素，均未达到持久、显著的效果[15]–[16]。克拉屈滨对SM各亚型均有疗效，剂量为0.14 mg·kg^−1^·d^−1^，连用5 d，每4～12周重复1次。法国报道68例成人肥大细胞增多症（mastocytosis，MC），其中惰性MC 36例（皮肤肥大细胞增生症6例，ISM 28例，SSM 2例），AdvSM 32例（ASM 14例，SM-AHN 17例，MCL 1例），克拉屈滨单药治疗中位3.7个疗程，总反应率（ORR）72％，惰性MC和AdvSM的ORR分别为92％和50％，克拉屈滨单药治疗MCL比其他AdvSM亚型的疗效差[17]。③靶向治疗，如TKI。因为KIT基因在MCL中的高突变率，伊马替尼、达沙替尼、米哚妥林、阿伐替尼等TKI也被用于MCL的治疗。伊马替尼对野生型、KIT V560G和F522C位点突变都有活性，但对KIT D816V突变耐药[18]。达沙替尼对野生型及KIT D816V位点突变均有抗肿瘤活性[19]。米哚妥林是一种小分子TKI，对于合并KIT D816V突变的肿瘤性肥大细胞有很强的抑制作用[20]–[22]。Gotlib等[22]采用米哚妥林100 mg每日2次治疗16例MCL患者，ORR 50％，中位无进展生存时间11.3个月。米哚妥林在美国被批准用于ASM、SMAHD和MCL的治疗[4],[22]。克拉屈滨联合米哚妥林的体外研究表明，两药联合对合并KIT D816V位点突变的肿瘤性肥大细胞疾病有很强的协同治疗作用[23]。阿伐替尼是一种高选择性的TKI，可高效抑制血小板衍生生长因子受体α（PDGFRA）和KIT D816V。体外研究发现，阿伐替尼对KIT D816V的抑制效果优于米哚妥林[24]。一项阿伐替尼治疗AdvSM（包括MCL）的Ⅰ期临床研究显示，经中位治疗9个月，18例可评估患者的ORR为72％，56％的患者达到完全或部分缓解。患者的肥大细胞负荷和D816V突变等位基因比例持续降低[25]。2021年6月，美国FDA批准阿伐替尼治疗AdvSM。目前，对病情进展迅速和（或）对TKI、克拉屈滨耐药的患者，诱导治疗推荐联合化疗[26]。④异基因造血干细胞移植（allo-HSCT），虽然目前临床数据非常有限，但allo-HSCT或许是治疗MCL相对有效的方法[27]–[28]。国际多中心回顾性研究显示，57例SM患者接受allo-HSCT，3年总生存（OS）率57％，其中SM-AHN 38例，ASM 7例，MCL 12例，OS率分别为74％、43％和17％。所以，年轻、一般状况可、有合适供者的MCL患者，应该考虑行allo-HSCT，清髓性预处理方案要优于非清髓性预处理方案[28]。我们报道的患者应用TKI+克拉屈滨+阿糖胞苷联合诱导化疗，并应用地塞米松、氯雷他定抗组胺治疗，1个疗程后达形态学CR、MRD阴性、KIT D816H突变未测出，组胺释放症状也得到缓解。继续TKI+克拉屈滨+阿糖胞苷巩固治疗1个疗程后尽早完成了allo-HSCT，移植后达沙替尼维持治疗。

目前尚无针对MCL独立的预后分层系统，仍参考SM或者AdvSM的预后分层。根据2016年WHO分型标准对SM患者进行预后分层提示MCL预后极差[2]。对SM分子生物学的进一步研究发现，S/A/R基因突变为AdvSM预后不良的独立危险因素，根据基因突变的数量可将AdvSM分为三个风险组[29]。2019年又提出了AdvSM的MARS预后评分系统[30]，在该评分系统中，HGB<100 g/L、PLT<100×10^9^/L、年龄>60岁、S/A/R中1个基因突变各积1分；S/A/R中≥2个基因突变积2分。低危组：0～1分，中危组：2分，高危组：3～5分。且相比WHO和S/A/R预后分层体系，MARS预后评分系统的预测指数最高。我们报道的该例患者根据MARS积分系统，积2分，为中危组。此外，有研究报道SM患者中血清sCD25水平升高（>1902 pg/ml）者生存更差，较高的sCD25不管对ISM还是AdvSM都是独立的预后不良因素[31]，本例患者治疗前sCD25为30 306.78 pg/ml，提示预后较差。

综上所述，MCL临床上极为罕见，可累及多器官系统，尤其本例患者发病初期合并嗜酸性粒细胞增多，增加了诊断的困难，容易误诊。因此，该病例提示我们若患者发生不明原因的反复发作性过敏反应、皮肤潮红、发热、脾大等症状，且合并嗜酸性粒细胞增多时，需开阔思路，综合生化、形态学、免疫组化、流式细胞学、分子生物学等多种检测手段来帮助诊断，从而实现精准治疗。TKI加联合化疗序贯allo-HSCT或许是目前MCL最好的治疗选择之一，但其远期疗效仍待观察，新的药物和治疗方案也还需进一步探索。
